# Crosstalk between the Intestinal Virome and Other Components of the Microbiota, and Its Effect on Intestinal Mucosal Response and Diseases

**DOI:** 10.1155/2022/7883945

**Published:** 2022-09-27

**Authors:** Njinju Asaba Clinton, Sodiq Ayobami Hameed, Eugene Kusi Agyei, Joy Chinwendu Jacob, Victor Oyewale Oyebanji, Cyril Ekabe Jabea

**Affiliations:** ^1^Health and Empowerment Foundation, Cameroon; ^2^Mbonge District Hospital, Cameroon; ^3^University of Buea, Cameroon; ^4^Univ Lyon, Université Claude Bernard Lyon 1, 69100 Villeurbanne, France; ^5^Faculty of Pharmacy and Pharmaceutical Sciences, Kwame Nkrumah University of Science and Technology, Ghana

## Abstract

In recent years, there has been ample evidence illustrating the effect of microbiota on gut immunity, homeostasis, and disease. Most of these studies have engaged more efforts in understanding the role of the bacteriome in gut mucosal immunity and disease. However, studies on the virome and its influence on gut mucosal immunity and pathology are still at infancy owing to limited metagenomic tools. Nonetheless, the existing studies on the virome have largely been focused on the bacteriophages as these represent the main component of the virome with little information on endogenous retroviruses (ERVs) and eukaryotic viruses. In this review, we describe the gut virome, and its role in gut mucosal response and disease progression. We also explore the crosstalk between the virome and other microorganisms in the gut mucosa and elaborate on how these interactions shape the gut mucosal immunity going from bacteriophages through ERVs to eukaryotic viruses. Finally, we elucidate the potential contribution of this crosstalk in the pathogenesis of inflammatory bowel diseases and colon cancer.

## 1. Introduction

The role of the microbiota in controlling mucosal immunity and diseases has vastly gained interest in recent years. Several studies have revealed connecting links between altered microbiota (dysbiosis) and disease, thereby necessitating in-depth studies into these microbial communities. The intestinal mucosa is composed of a complex plethora of cells which via suitable interactions enable a tolerant immunological environment necessary for maintenance of homeostasis. The main components include intestinal epithelial cells, immune cells, microbiota, and metabolites [1]. The crosstalk between these constituents is necessary to create a balance in immune tolerance and protective immune response to self and non-self, respectively. Conversely, an alteration in these constituents is associated with inappropriate immune response and may give rise to diseases or abnormalities. The maintenance of mucosal barrier is quite challenging as it is exposed to many affected by both genetic and environmental factors like food, toxins, drugs, and microorganism that can induce a damaging effect [2]. The intestinal epithelial cells comprise many subtypes which include enterocytes, goblet cells, Paneth cells, enteroendocrine, and M cells, distributed at various levels in the small and large intestine and have varying functional attribute [3]. These cells express different pattern recognition receptors including Toll-like receptors (TLRs), C-type lectin receptors, retinoic acid-inducible gene (RIG)-I-like receptors (RLRs), nucleotide-binding oligomerization domain- (NOD-) like receptors (NLRs), and absent in melanoma 2- (AIM2-) like receptors (ALRs) [4, 5]. In response to different PAMPs and specific cytokines, these cells have different effector functions. For example, Paneth cells produce antimicrobial peptides, goblet cells secrete mucus, and M cells are important in antigen uptake, phagocytosis, and transcytosis [6, 7]. Underneath the epithelial cell layer is the lamina propria which contains many immune cells (macrophages, dendritic cells, B cells, and T cells). The intestinal mucosa is very important in nutrient absorption (enterocytes) and establishment of both chemical and physical barrier against luminal contents. Furthermore, the epithelium encloses various lymphoid aggregates, the most important being Peyer's patches which are composed of B cell follicles and T cell areas important in adaptive immune response. These remain the main source of intestinal IgA that is one of the main effector molecules in adaptive immunity [2]. The induction of mucosal immunity takes place in Peyer's patches, and other lymphoid aggregates. Interestingly, the effector response following immune induction can be appreciated both at local and distant sites. This is very important as changes in mucosal immunity can be translated to systemic effect at distant sites. The various immune cells in the mucosa have specific phenotypes and functions. Majority of macrophages in the lamina express the CX3CR1 receptor and function mainly in the regulation of gut intestinal homeostasis via capturing and destruction of food and pathogenic antigens. They also exert an immune regulatory effect by secreting IL-10 which favors Foxp3+Treg polarization [8]. Similarly, different phenotypes of conventional dendritic cells are found in the lamina propria depending on their level of expression of CD11b and CD 103. The colon contains more of CD11b^−^CD103^+^ DCs in contrast to the small intestine containing CD11b^+^CD103^+^ DCs [2]. Both the conventional and plasmacytoid dendritic cells are important in the modulation inflammatory and adaptive response in the intestine. Another class of immune cells important in intestinal immune regulation are the innate lymphoid cells which are categorized into ILC1, ILC2, and ILC3 that activate Th1, Th2, and Th3/Th17 responses, respectively [9]. These cells respond to various immunomodulatory signals from microbes, metabolites, and dietary antigens. The signals stimulate epithelial secretion of various immunomodulatory and inflammatory cytokines important in intestinal homeostasis and inflammation, respectively [9]. Nonetheless, the homeostatic state of the intestinal mucosa depends on the balance between anti-inflammatory (Tregs) and proinflammatory T cells (Th1, Th2, Th3/Th17) [2]. Intestinal homeostasis is greatly affected directly or indirectly by the diversity of the microbiota. This diversity is in turn affected by many factors from birth, including the method of delivery, diet, drugs, infections, and genetic factors. The microbiota consists of the bacteriome, virome, mycobiome, and even some parasites. However, most studies have been done on the characterization and effect of the bacteriome on mucosal immunity and disease [10]. Studies have shown that the diversity of the bacteriome has an influential effect on the intestinal immune tolerance, mucosal immunity, and diseases. This is due to the release of various metabolites from both in the small and large intestine that helps in the induction of immune tolerance. In the small intestine, protective bacteria like the Firmicutes and proteobacteria produce metabolites like pyruvate, lactate, branched chain fatty acids, and amino acids from starch, lipids, and proteins, respectively. These metabolites stimulate the maintenance of intestinal mucosa integrity and induce intestinal immune tolerance. Likewise, in the large intestine, *Bacteroides* produce short chain fatty acids, tryptophane, proline, and other factors important for maintenance of colonic mucosal integrity [2]. These metabolites are important in the modulation of proinflammatory responses and the induction of regulatory T cells. For example, short fatty acids produced by commensals from dietary fibers increase the frequency of Tregs [11] and inhibit histone deacetylase thereby promoting the maintenance of epithelial cell integrity and tolerance to bacterial and dietary antigens [12].

Despite more elaborate studies on the bacteriome, the characterization and role of the virome in the mucosa are still at infancy. However, recent advances in sequencing and metagenomic analysis have significantly improved the study and characterization of the virome [13, 14]. Interestingly despite the presence of the virome in different parts of the human body, the gut virome makes up the bulk of the human virome that has been shown to colonize the gut epithelium and is the most studied [15]. Recently, there has been growing evidence associating the virome with host physiology and disease development. For example, human endogenous retroviruses have been shown to influence placenta development and enhance antiviral immune response. Furthermore, enteric RNA viruses have been shown to mimic the beneficial function of commensal bacteria in the gut [16]. Nevertheless, data on virome trans-kingdom interaction, host intestinal immunity and disease development is still not clear. Thus, the aim of this review is to describe the origin and characteristics of the intestinal virome, virome trans-kingdom interaction, and its effect on mucosal immunity and intestinal disease.

## 2. Metagenomics of the Gut Virome

There exists on earth an estimated 10^31^ viral particles making them the most abundant entities on earth, with an estimated 10^9^ virus-like particles per gram of human feces [17]. Most of these viruses are identified as prokaryotic viruses which infect bacteria, but a great majority of these viruses are still unidentified. More so, gut virome of different individuals oftentimes yields novel viruses with only a small fraction of the ORFs corresponding to previously identified genes [17].Generally, the human body is inhabited by eukaryotic and prokaryotic viruses which infect human and bacterial cells, respectively. Research had historically been focused on the eukaryotic viruses because of their impact on human health. More recently, increasing evidence is showing that the prokaryotic viruses also have impacts on the human health through their interaction with the human symbiotic bacteria thereby shaping the bacterial communities in terms of structural and functional composition in regions where there is high abundance of the bacteria such as the human gut [18]. The human virome simply refers to the collection of all the viruses infecting and/or cohabiting the human body [19]. Indeed, these viruses have also been recently associated with their own suffixes “ome” and “omics,” viz., the terms “virome” and “viromics,” referring, respectively, to the collection of these viruses and the study of their genomes [18].To facilitate the study of the virome, the first step is to identify the viruses in their complex communities; however, this has proven to be problematic owing to the fact that these viruses lack a universal marker such as the 16S rRNA of the bacterial genome [20]. Furthermore, the annotation of the human virome has been largely impacted by high diversity of the viral genomes found in the different anatomical sites which could have ssRNA, dsRNA, ssDNA, or dsDNA genomes(Table 1). Nevertheless, the recent advancement in the next-generation sequencing and metagenomic data analysis has greatly facilitated the understanding and annotation of the virome [9].

**Figure 1:**
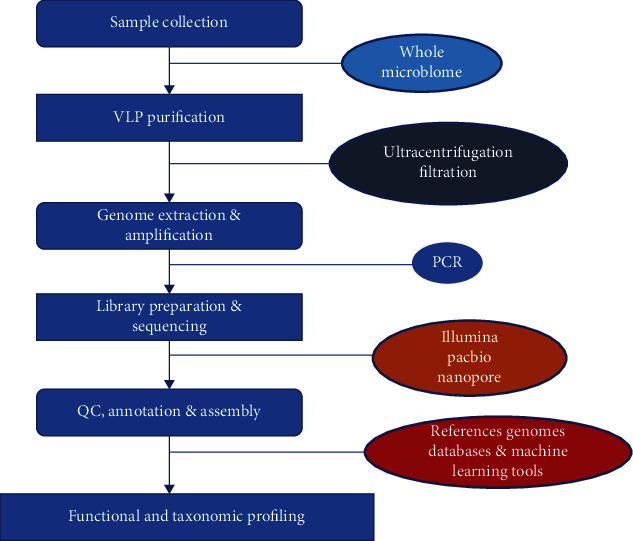
Viral metagenomic workflow. A schematic representation of the steps and processes involved in the isolation, identification, and analysis of the virome. Following sample collection, the sample is purified through a series of steps involving ultracentrifugation and filtration to retain the virus-like particles (VLPs). This is followed by extraction steps to isolate the genomes of the VLPs which are then amplified by PCR, utilized for library preparation and sequenced using sequencing technologies such as Illumina, Pacbio, or Oxford nanopore technology. The sequencing generates reads—short, long, or ultra-long—which are quality controlled, annotated, and assembled using different databases and machine learning tools. Finally, the identified VLPs are then subjected to taxonomic and functional profiling to answer key biological questions.

Although many pathogenic viruses causing diseases in the human gut have been characterized and reported long time ago, the concept of the human gut virome is paradoxically recent [21]. The research focusing on the gut virome which encompass the viral component of the gut microbiome is generally lagging. Nevertheless, the study of the gut virome often starts with the purification of the viral particles, the removal of other cells, and the elimination of free-floating nucleic acids in a series of steps involving filtration, centrifugation and enzymatic reactions (Figure 1). This is followed by the extraction and the amplification of the viral nucleic acids. This entire process is complicated by the fact that intracellular viruses are neglected, by the difficulty to simultaneously amplify the different types of viral genomes and by the lack of targeted conserved viral elements. Furthermore, research in the human gut virome is further retarded by the limited viral databases and bioinformatic tools [22].More recently, several emerging methods have been employed in virome isolation, purification, and quantification, each of which has its peculiar advantage and limitations. For example, the traditional sampling method, e.g., using a 2-micron filter, can be biased towards isolating the most abundant virome species in the compartment of interest. Similarly, the caesium chloride (CsCl) gradient ultracentrifugation purification technique can be biased towards species with atypical buoyancy and specific phage type depending on how the method has been performed, although this technique yields a very pure virome isolate. Furthermore, the epifluorescence microscopy technique for viral quantification can sometimes result in underestimation of the virus-like particles in a sample. Owing to these drawbacks, automated extraction methods are now employed for viral detection, often combined with qPCR and droplet-based digital PCR because these offers a higher sensitivity and allows a high throughput work capacity [23]. The biases and challenges associated with viral metagenomics had been extensively covered in other reviews [24–26] and as such would not be explored deeply in this review. The human gut is intricately inhabited by a community of viruses forming the “virome” part of the microbiota. A very large fraction of the gut virome is represented by the bacteriophages and the endogenous retroviruses, although most attention on virology has generally focused on the pathogenic animal bacteria [9]. Overall, the human gut virome encompasses the prokaryotic viruses (bacteriophages), the endogenous retroviruses, and the eukaryotic viruses. The bacteriophages remain by far the largest part of the gut virome, representing over 90% of the total viruses in the gut [21]. These prokaryotic bacteria-infecting bacteria are about 10-fold higher the gut bacteria and with which they interact, thereby, largely modifying the composition of the bacterial microbiota [27]. These interactions occur through the lysis of the bacteria resulting in the generation of new phage particles, or through the integration of the phage genome into the bacterial genome. This results in the production of new phages and changes in bacterial fitness, phenotype, or bacterial-host interaction; conferment of resistance genes, changes in bacterial ability to produce toxins or increased bacterial energy yield. Hence, bacteriophages are literally referred to as bacterial parasites or viruses of bacteria [21, 27]. There are approximately 10^15^ bacteriophages occurring in the human gut and the majority of these particles contain the DNA genomes. In fact, of the total DNA viruses that can be matched to an annotated genome database, 99% represent the bacteriophages and the remaining 1% are the eukaryotic animal viruses [9]. Transmission electron microscopic studies and next-generation sequencing analysis have revealed that the genomes of most of the gut bacteriophages belong either to the dsDNA viruses of the order *Caudovirales* which encompasses the families *Siphoviridae*, *Podoviridae*, and *Myoviridae*, or the ssDNA viruses which generally belong to the family *Microviridae.* In addition, the order *Caudovirales* has been recently expanded to include the families *Ackermannviridae* and *Herelleviridae* [9, 17, 18]. The *Microviridae* family consists of a group of viruses with a single-stranded circular DNA genome and is subdivided into three groups based on structural and genomic differences. This includes the microviruses (genus *Microvirus*) that exclusively infect *Enterobacteria*; the gokushoviruses (subfamily *Gokushovirinae*) that infect obligate intracellular bacteria of the genera *Chlamydia*, *Bdellovibrio*, and *Spiroplasma*, and a more recently classified viruses of the sub-family *Alpavirinae* which are generally prophages residing in the genomes of the bacteria of the genera *Prevotella* and *Bacteroides* [28].However, an unclassified group of bacteriophages with dsDNA known as the crAssphage has been found to be abundant in about 73% of human fecal metagonome and is predicted to infect the *Bacteroides*. Furthermore, the crAssphage-like genome was shown to be present in most of the old and new-world primate samples in a highly divergent but collinear manner, thus suggesting a new phage family with evolutionarily stable genomes for millions of years [9, 18, 29]. The endogenous retroviruses (ERV) forming part of the virome are similar to the present-day exogenous retroviruses but have been integrated in the host genome and are being transferred from generation to generation; this makes up about 8% of the human genome. For example, the syncytin protein that plays role in the development of the human placenta was derived from the *env* gene of ERV. The ERVs have also been predicted to play role in human evolution. These viruses have accumulated sufficient mutations over time which has rendered them defective and non-pathogenic. However, there exist some ERVs with the potential to assemble to a full viral element which are capable of triggering the immune response through the PRR [30]. The activated ERVs can also lead to cancer as many cancers have been linked to the transcriptional activation of the human ERVs. This can trigger insertional mutagenesis and chromosomal rearrangements which can influence cellular expression of genes [31]. In addition, the ERVs have evolutionarily been shown to have shaped the interferon response pathway and different lineage-specific-ERVs had dispersed diverse interferon-inducible enhancers independently in the mammalian genomes [32].The eukaryotic viruses forming part of the gut virome consist of all other RNA or DNA viruses apart from the bacteriophages and the ERVs [9]. These are however relatively fewer than the bacteriophages [19]. These viruses are capable of infecting the human host cells, the intestinal fungi, and parasites or are viruses just passing through the gut like the plant viruses [22]. Many of these viruses have been clearly established to cause acute or chronic intestinal disorders such as gastroenteritis and diarrhea. For example, diarrhea is known to be frequently caused by Norwalk, Rotavirus, and Enterovirus in human and more recently, viral families such as *Adenoviridae*, *Picornaviridae*, *Reoviridae* which were previously thought to be non-pathogenic have been implicated as a cause of diarrhea in children following advanced metagenomics [33, 34]. There is limited information regarding the beneficial role of the eukaryotic viruses in health. Nonetheless, it was reveled in a study that an enteric RNA virus such as the Murine norovirus (MNV) can replace the role of the beneficial intestinal bacteria. Here, the MNV infection of antibiotic-treated or germ-free mice resulted in the restoration of the intestinal morphology without inducing overt inflammation [16]. Furthermore, viruses belonging to the families *Anelloviridae* and *Circoviridae* are frequently isolated in the human stool without pathology, indicating a probable commensal relationship of these viruses with the human gut [35]. In addition, sequencing of the fecal sample in healthy infants revealed the presence viruses of the families *Picobirnaviridae*, *Adenoviridae*, *Anelloviridae*, and *Astroviridae* and several species such as bocaviruses, enteroviruses, rotaviruses, and sapoviruses [19, 36].

Generally, there exist temporal dynamics in the gut virome but the variability of the gut virome is poorly studied. Nonetheless, the available studies have revealed a relatively stable intrapersonal virome composition and highly variable interpersonal intestinal virome communities. One study depicted that over 80% of the gut virome is retained in adult individuals over a period of 2.5 years for which the study was carried out [17]. This result was replicated recently in another where the viral composition was reported to be retained throughout the 26 months of study in terms of alpha diversity and total viral count which correlate with the bacterial microbiome [37]. Diet has been shown to play significant role in shaping the gut virome. Individuals with the same diet were found to be relatively similar in the composition of their virome which was stable over time and with the highest variance being due to interindividual variability [38]. The large interpersonal variability in the gut virome has been linked to environmental influence rather than genetic factors as shown in monozygotic twin studies that co-twins do not share more virotypes than unrelated individuals as they age and that the bacterial microbiome largely determines the virome [39]. Birth mode has also been shown to influence the gut virome composition as revealed in a study that the virotype largely correlates with birth-mode following the comparison of the virome in infants born by spontaneous vaginal delivery and caesarian section at age 1 year after birth [40]. In addition, the virome has been shown to colonize the gut shortly after birth and varies at different time points in the first month of life through 2-3 years of age from when the virome becomes stable over time. In a study, the meconium screened immediately after birth contained no viral particles but when the same infant was screened after one week, the feces contained 10^8^ virus-like particles [18, 22, 41, 42].

## 3. Functional Landscape of the Gut Virome

The functional significance of the gut virome was less well established not until more recently when new studies are revealing evidence of the functional attributes of the virome in the intestinal environment. The gut-associated phage has been reported from recent metagenomic surveys to encode genes that performs beneficial functions to the intestinal bacteria ranging from bacterial virulence, host bacterial adaptation to the intestinal environment, and maintaining host microbiome stability and community resilience [43]. For example, it was shown in a previous study that cryptic prophages had a significant contribution to the resistance of bacteria to sublethal concentrations of quinolone and beta-lactam antibiotics. In addition, these prophages also offered the beneficial roles in withstanding oxidative and acid stress, influencing biofilm formation, and increasing bacterial growth [44]. Furthermore, an unrelated study revealed the role of phages in serving has a reservoir for beneficial genes which could be the source of important genes to the gut microbiome in the face of depletion resulting from antibiotic stress. In this study, it was shown that antibiotic treatment resulted in the enrichment of phage-encoded genes that confers resistance not only against the administered antibiotics but also other antibiotics. It was also revealed that this antibiotic treatment increases phage-bacteria interactions which enhances gene exchange networks that facilitate host colonization, bacterial growth, and adaptation [45].

**Figure 2:**
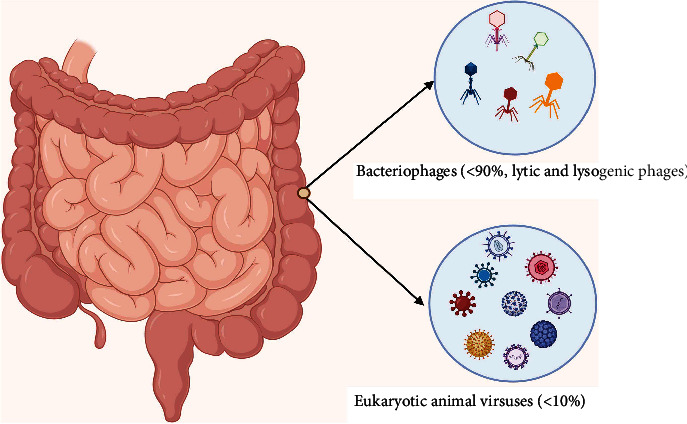
Figure illustrates the two main compositions of the human intestinal virome (bacteriophages and eukaryotic viruses). The bacteriophages present the most abundant and can be subdivided into lytic and lysogenic phages depending on the infection outcome in bacteria.

Indeed, the phage-encoded antibiotic resistance genes which are highly diverse mobile genetic elements could undoubtedly contribute to the emergence and spread of antibiotic resistance within and outside the human gut. These resistance genes could be transferred through the establishment of networks that facilitate gene exchange within the microbiome community, most especially via the process of transduction [43]. Overall, it can be concluded that the phage has a diverse functional repertoire within the gut environment; these are majorly of beneficial roles to the gut bacterial community but could be of negative influence on the human gut health. The distribution and some examples of the intestinal virome are summarized in Figure 2 and Table 2, respectively.

## 4. Effect of the Virome on the Intestinal Mucosal Immunity

Generally, there exists a dynamic equilibrium between the intestinal immune system and the gut microbiota including the virome and this interaction influences both health and disease through the modulation of the mucosal immune system. The virome remains a potent regulator of the intestinal immunity in terms of the balance between homeostasis and inflammation as these resident enteric viruses are known to continuously stimulate the gut immune system without overt symptoms [19]. Indeed, the effect of the virome on the intestinal mucosal immunity can be orchestrated either by their direct interaction with the host cells or indirectly through a trans-kingdom interaction with the other microbiota. Since bacteriophage composition has been shown to shape the gut bacterial communities and intestinal diseases have been linked to bacterial dysbiosis, the gut virome can indirectly influence the mucosal immunity through their interactions with the intestinal bacteriome. Hence, by shaping the gut bacteriome, the virome indirectly influence the intestinal physiology as well as the development and function of the gut immune system [50]. For example, it was shown in a study that changes in the virome taxonomic composition with the expansion of the *Caudovirales* bacteriophages correlated with bacterial dysbiosis in Chron's disease and ulcerative colitis patient compared to control [51]. It has also been shown that phages adhere with the mucus on the intestinal epithelial surface to form a protective barrier which prevents bacterial infection/translocation across the intestinal mucosa. This interaction occurs via the binding of the Ig-like domain present on the capsid of the phages with the variable glycan portions of the mucin glycoprotein component of mucus [52]. It is equally possible that the bacterial cell wall and/or lytic product released following bacterial lysis secondary to phage infection of the bacteria can be sensed by the pattern recognition receptors (PRRs) on the intestinal epithelial cells or resident immune cells, thereby triggering an immune response that may influence intestinal homeostasis and immunity [50]. The intestinal virome can also intrinsically modulate the gut mucosal immunity. Since viruses are intracellular organisms, they require infection of the host intestinal cells in order to propagate their life cycle; this is especially important for the eukaryotic viruses but there are evidences that the bacteriophages may also interact directly with the host cells [9]. The intestinal epithelial cells possess the PRRs which are sensors for these viral particles to induce an immune response. In addition, the submucosal DC and macrophages also play role in sensing the enteric viruses following translocation of the viruses to the submucosa [9, 53]. It was also shown that bacteriophages were able to cross different epithelial barriers of different tissue origin [54] and orally administered *E. coli* phage was reported to translocate to distal tissues including the spleen with the induction of both innate and adaptive responses [55]. Furthermore, it is possible for prophages to go beyond the gut and produce the encoded bacteriophage which can then be detected by the host immune cells. It was shown in a study that bacteriophages produced from *Pseudomonas aeruginosa* was internalized by DC, macrophages, and B-cells to induce type-I interferon responses thereby facilitating infection by related bacteria [56]. Therefore, these are valuable evidences that both eukaryotic viruses and the bacteriophages can interact with immune cells at the intestinal mucosa and even beyond. Many intracellular and cytosolic receptors present on the intestinal epithelial cells and the innate immune cells can detect the viral genomes. These include the viral RNA sensors, viz, TLRs such as TLR3, TLR7, and TLR8; NLRs such as NLRPs; RLRs such as RIG-I and MDA-5; and the DNA sensors such as the endoplasmic TLR9 and the cytoplasmic cGAS-STING pathway, all of which can sense different PAMPs including the viral genomes. The recognition of eukaryotic viruses by these receptors is essential to control infection. The exact mechanism of recognition of bacteriophages is not well known but TLR3/7/8 can detect RNA transcripts from bacteriophages [53]. Activation of these sensors triggers signaling pathways that result in the downstream production of NF-*κ*B, IRF3, and IRF7 which in turn induce the production of antiviral mediators such as type-I interferons, cytokines (e.g., IL-1 and IL-6), and chemokines (CXCL8, CXCL10). These mediators, constantly produced following the recognition of bacteriophages, act on intestinal epithelial cells and immune cells thereby stimulating a tonic antiviral intestinal environment which prevent pathogenic viral colonization of the intestine [19]. In addition, it has been suggested that chronically resident viruses in human healthy tissues such as Herpesviruses, Poliomaviruses, Adenoviruses, Papillomaviruses, Hepatitis B and C viruses, and HIV can induce acute or chronic infections which can prevent the colonization of the intestine by other pathogenic bacteria and viruses. Experimental mice model latently infected with Herpesviruses was resistant to infections from *Listeria monocytogenes* and *Yersinia pestis*; this was linked to latency-induced basal activation of the innate antiviral immunity via the production of antiviral cytokines and the activation of macrophages [49]. However, chronic viral persistence may also bring about a reduced host intestinal immunity and increased susceptibility to infection. This can occur due to damage to epithelial barrier which can facilitate infection by other pathogens as well as chronic immunosuppression which increases the susceptibility of the host leading to rapid translocation of pathogens across the intestinal barrier, thereby causing intestinal inflammation and/or systemic infection. For example, pathogenic AIDS following SIV infection in non-human primates was associated with the expansion of the gut virome, therefore suggesting the contribution of enteric viral infection to AIDS enteropathy [57, 58].

## 5. Crosstalk between the Virome and the Bacteriome in the Intestinal Mucosa

There exist extensive interactions between the eukaryotic viruses and the commensal bacteriome in a manner that influence not only viral infectivity but also host immunity. Influence on the viral infectivity may however be positive, enhancing infection or negative, impairing infection. This positive effect can occur following direct physical interaction of the viruses such as poliovirus and reovirus with the commensal bacteria to enhance viral infection. For example, it has been shown that poliovirus binds to the surface polysaccharide of commensal bacteria; this facilitates the binding of the virus with cellular receptors thereby enhancing the viral stability and cellular attachment. Recent studies have also shown that poliovirus co-infection of mammalian cells is enhanced by commensal bacteria which improve the genetic recombination of the virus [59–61]. It was shown in a study that antibiotic treatment reduced both the severity and pathogenesis of reovirus in mice whereas a more severe disease develops in the absence of antibiotic treatment, suggesting that the bacterial microbiota enhances reovirus pathogenesis [62]. The gut commensal microbiota has also been shown to be involved in mouse mammary tumor virus (MMTV) persistence and transmission to pups through milk. It was initially known that TLR4 and IL10 play role in MMTV persistence and mammary transmission. Much later, it was depicted that MMTV persistence enhanced by TLR4 and IL10 is microbiota dependent. It was experimentally demonstrated that germ-free and antibiotic-treated mice do not transmit MMTV to their offspring. In a mechanistic fashion, it was revealed that the virion attaches to the bacterial LPS to induce TLR4/IL10 signaling that facilitate viral persistence and transmission [61, 63]. Conversely, the gut bacteriome may serve to impair successful viral infectivity and pathogenesis. For example, it has been shown that probiotics, specifically *Lactobacillus*, reduces viral diarrhea induced by rotavirus. Furthermore, a study revealed that soluble factors from the commensal bacteria of the genera *Lactobacillus* and *Bacteroides* were able to inhibit rotavirus infection of intestinal epithelial cells *in vitro* by modulating surface glycan expressions which impairs rotavirus attachment to the intestinal cells [64]. Commensal bacteria have been reported to modulate influenza virus and lymphocytic choriomeningitis virus (LCMV) susceptibility. It was depicted that antibiotic-treated mice developed a severe bronchiolar damage and presented higher mortality after mucosal influenza challenge while also showing a more delayed viral clearance following mucosal influenza virus and systemic LCMV challenges. This was linked to defective innate and adaptive immune response in the antibiotic-treated mice with an impaired type-I and type-II IFN responses and downregulated protective antiviral genes in macrophages. It was concluded therein that the commensal bacteria maintains a tonic immune-stimulation that lowers the activation threshold for innate responses against the viruses [65]. Overall, it can be inferred that the type of interaction between eukaryotic viruses and the gut bacteriome is dynamic and largely dependent on the type of virus in question; this in turn influences the impact on the host gut health and disease.

### 5.1. Crosstalk between Bacteriophages and Intestinal Bacteria

**Figure 3:**
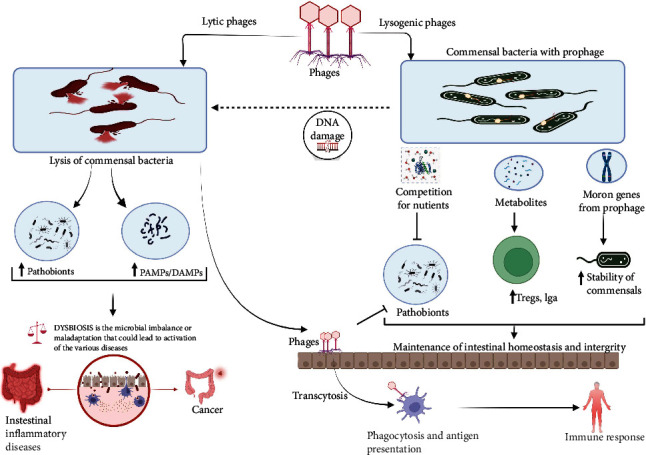
This figure illustrates the role of lytic and lysogenic phages in the induction and protection of intestinal dysbiosis and inflammation. The lysis of bacteria by lytic phages leads to the release of pathogen-associated molecular patterns (PAMPS) and danger-associated molecular patterns (DAMPS) which trigger the release of proinflammatory cytokines leading to intestinal inflammation and dysbiosis. However, phages released from lysed bacteria can bind to the intestinal mucosa and protect against pathobionts. Phages can also cross the mucosal barrier by transcytosis and induce local and systemic immune response. On the contrary, commensal bacteria protect the intestinal mucosa from pathobionts and helps in the maintenance of intestinal homeostasis and integrity via competition for nutrients with pathobionts, induction of Tregs, IgA. Nevertheless, following stress factors which lead to DNA damage these prophages can become lytic triggering inflammation.

The presence of commensal bacteria like Firmicutes, proteobacteria, and *Bacteroides* are fundamental for the development of mucosal immune tolerance and maintenance of mucosal integrity. Contrastingly, a reduction of these protective commensals is associated with the development of intestinal inflammatory diseases. The population and diversity of the bacteriome are affected by many intrinsic and extrinsic factors, among which are the bacteriophages. There are approximately 10^15^ bacteriophages in the human gut. The interaction between the phage and the bacteriome is known to be species specific [66], although recent evidence suggests that phages can promiscuously interact with many species of the bacteriome [67, 68], thus increasing the capacity of phages to infect a wide variety of gut bacterial species. This is important because via infection of many bacteria species, phages are able to modulate the bacteriome diversity. The life cycle of phages affects microbiome diversity, depending on whether it is lytic or lysogenic. The lytic life cycle is more detrimental to commensals, as it is characterized by breakdown of bacteria and release of numerous PAMPs and DAMPs [69]. On the contrary, the lysogenic life cycle involves the formation of a prophage. The phages are either integrated in the bacteria chromosomes or plasmid where they stay in a dormancy. They contain many repression genes and are capable of passive replication in the bacteria genome. Nevertheless, in the presence of stress factors, prophages can be skewed towards the lytic cycle in a process called prophage induction. The molecular mechanism underlying prophage induction is based on DNA damage which destabilizes the repressors of prophage induction [66]. Some of the triggers of DNA damage include quinolones [70], bacteria metabolites like nitric oxide [71], bile salts [72], and others. The effect of prophage induction can either be detrimental or beneficial depending on whether it is activated in commensal or pathogenic intestinal bacteria. This is because commensal bacteria are vital in the development of IgA plasma cells, CD4 T cells, lymphoid follicles, and invariant natural killer T cells [73–75]. Interestingly, it has been shown that Firmicutes and proteobacteria harbor the bulk of the lysogenic prophages [76]. These prophages prevent infection of the commensals by other lytic and lysogenic phages via super-immunity exclusion [77], a phenomenon whereby existing viral infection protects against reinfection or infection from closely related viruses. This is crucial, as lysis of these commensals following prophage induction decreases the microbiome diversity and predisposes to dysbiosis. Thus, emphasizing the role of phages in the modulation of bacteriome diversity and induction of dysbiosis, dysbiosis can be characterized by impaired mucus secretion, inflammation, loss of mucosal integrity, and increased immune cell infiltration which are typical of diseases like Crohn's disease and ulcerative colitis. An understanding of the molecular mechanisms that trigger prophage activation in protective commensal bacteria could be revealing in the development of protective measures against dysbiosis and inflammatory bowel diseases. Furthermore, the lysis of bacteria by phages increases the release of PAMPs and DAMPs that trigger the release of proinflammatory cytokines in the intestinal environment. This inflammatory environment affects the metabolic activities of bacteria, thereby affecting the release of essential metabolites like short chain fatty acids important for maintaining immune tolerance and mucosal integrity [78], consequently favoring inappropriate immune response to intestinal microbiome and tolerogenic antigens. On another note, the released phages can be trapped in the mucous layer via specific interactions. For example, T4 phages bind via their capsid proteins specifically to mucin in the mucous layer and provide protection against invading bacterial infection [79]. In addition, released phages can cross the mucosal barrier via transcytosis, trojan horse mechanism, or mucosal gap junctions (in cases of dysbiosis leaky gut) to the lamina propria and systemic circulation [80]. These phages are phagocytosed and presented by antigen presenting cells, thereby contributing to systemic innate immune response. Some bacteriophages downregulate eukaryotic immune responses against bacteria via the expression of auxiliary ankyrin repeats (ANKs) [81]. Studies have elucidated the importance of ANK in bacteria specie- and trans-kingdom interaction [82]. Furthermore, Ankyphage-infected bacteria have efficiently exhibited characteristics of eukaryotic immune evasion [81]. The main mechanisms described include inhibition of phagocytosis and downregulation of inflammatory responses [56, 81, 83]. In this light, phages favor bacterial survival; however, more studies are required on this subject as it can be exploited as a therapeutic strategy in dysbiosis. Phages also play an important role in bacterial evolution and virulence via horizontal gene transfer. It has been well illustrated that phages are capable of transferring virulent factors and antibiotic resistant genes between bacteria via horizontal gene transfer. Horizontal gene transfer contributes to increase genomic complexity and functionality in bacteria, as well as evolution of new pathogenic forms of bacteria [84]. Although these evolutionary changes are detrimental to the host, they are essential for bacteria adaptation and survival to environmental stress or changes that occur over time. Phages also contain moron genes. The role of phage moron genes, which are genes present but not directly beneficial to the prophage, is capable of modifying bacteria phenotypes [85]. These genes indirectly increase the survival of prophages by prolonging the survival of their host cells. Moron genes are acquired by horizontal gene transfer and usually contain their own promoters and terminator sequences essential for expression in prophage. These genes have been shown to increase fitness and virulence in different species of bacteria via different mechanisms [85]. This is of great interest because the moron genes contain conserved clusters present in specific bacteria species. Hence, optimizing studies on the use of these moron genes in prolonging the fitness and stability of various protective commensals could pave new avenues in the management of dysbiosis. The bacteriome represent a site for phage replication and survival. However, phages have a significant role in the determination of bacteriome diversity which is paramount for maintenance of gut mucosal tolerance and integrity. The crosstalk between the virome and the bacteriome is summarized in Figure 3. Exploring this crosstalk can be s key in the management of inflammatory bowel diseases and control of intestinal pathogenic bacteria. Further understanding of metagenomic characterization of commensal prophage, as well as the molecular mechanisms triggering prophage induction, could help in the design of phage therapy against inflammatory bowel diseases. Also, target lytic phage therapy against pathogenic bacteria in patients with dysbiosis could also be exploited.

### 5.2. Crosstalk between Endogenous Retroviruses and Bacteriome

The human endogenous retroviruses (HERVs) contain long terminal repeats capable of influencing neighboring genes fundamental in the development of inflammatory bowel diseases and cancer. Few studies have described the distribution and effects of HERVs on the intestinal bacteriome and mucosal immunity. Nevertheless, notable disparities in the distribution and diversity of HEVs between patients with inflammatory bowel diseases and healthy individuals have been well elaborated [86].

**Figure 4:**
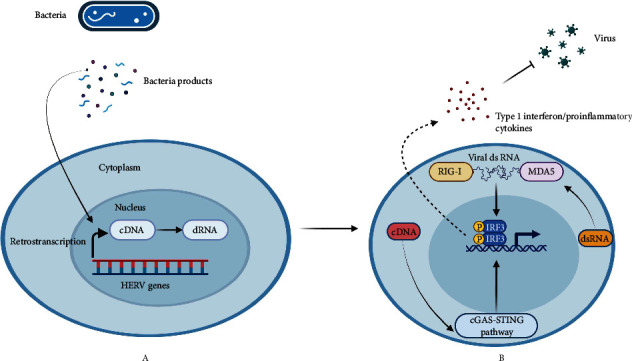
An illustration of retro-transcription of HERV (human endogenous retroviruses) or endogenous retroelements following stimuli released from commensal bacteria (a). Retro-transcription of human endogenous retroviruses (HERV) lead to the formation of cDNA and dsRNA that is released into the cytoplasm. The presence of cytosolic cDNA and dsRNA initiates a protective antiviral immune response via the activation of cGAS/STING and RIG-1/MAV pathways (b).

This coincides with the differences in the distribution of the bacteriome between Crohn's disease (CD) patients and healthy individuals [87]. Hence, it will be very interesting to decode the interplay between intestinal bacteriome and HERVs in both diseased and healthy patients. Nonetheless, there are no elaborate studies demonstrating this relationship, which can be of importance in the modulation and activation of intestinal mucosal immunity. As stated earlier, the HERVs express envelop proteins called syncytins that are important in inducing maternal-fetal immune tolerance. This includes the HERV-FRD env (Syncytin-2) and HERV-W env (syncytin-1) which both have immunomodulatory functions [88, 89]. The expression of both subgroups of proteins is reduced in patients with colonic inflammation and CD compared to healthy individuals [86]. This therefore suggests HERV could have an immune-modulatory role in maintaining bacteriome diversity and intestinal mucosa tolerance. Unfortunately to the best of our knowledge, no study has effectively demonstrated this phenomenon in the intestine. It was previously shown that the gut microbiota induces type-1 interferon antiviral response which protects against viral infections. The underlying molecular mechanism was linked to the activation of viral sensors which trigger type-I interferongenes [90]. However, the underlying triggering factors involved in the activation of viral sensors were still obscured. Nevertheless, recently it was illustrated that the skin bacteriome (Staphylococcus epidermis) via specific factors is capable of triggering retro-transcription of endogenous retroviruses that lead to the activation of the cGAS-STING pathway. The activation of cGAS-STING pathway (Figure 4) triggers the stimulation of Interferon-I stimulating genes that elicit a good antiviral response, and homeostatic T cell response to the skin bacteriome [86]. In this study, the presence of lipoproteins and teichoic acid present in staphylococcus epidermis triggered the activation of TLR2 signaling that reactivates HERVs retro-transcription. Other viral sensors activated by endo retroviral transcripts include RIG-1, MDA, TLR3, and TLR9 which all trigger the activation of interferon stimulating genes and antiviral immunity [91]. Furthermore, Endo-retroviral envelope [92, 93] and gag proteins [94, 95] are linked with the interference of both viral entry and replication. The outcomes of these studies are quite remarkable as it illustrates the effect of some protective responses that could be obtained from endo-retroviral reactivation by the bacteriome. Although there are differences in the biodiversity of the bacteriome between the skin and the intestine mucosa, there are still possibilities of discovering similar findings in the intestine. This is because as elaborated earlier, there is a distinct difference in diversity of HERV expression in patients with inflammatory bowel disease and healthy patients. An understanding of the crosstalk between the HERVs and the bacteriome could be paramount in the induction of effective protective immune response against viral diseases and maintenance of gut mucosal homeostasis. Hence, we recommend further studies on the crosstalk between the bacteriome and HERVs at the intestinal mucosa. This can lead to interesting findings that can be translated in the management of intestinal diseases associated with dysbiosis and intestinal viral diseases.

## 6. Crosstalk between the Virome and Mycobiome, and Its Effect on the Gut Immune System

The mycobiome is the fungal community of the microbiome and it is known to be less abundant and diverse than other microbiome constituents such as the bacteriome as it makes up only 0.01-3% of the gut microbiome [96]. The fungi genera detected in the microbiome include *Candida*, *Saccharomyces*, *Fusarium*, *Debaromyces*, *Penicillium*, *Galactomyces*, *Pichia*, *Cladosporium*, *Malassezia*, *Aspergillus*, *Cryptococcus*, *Trichosporon*, and *Cyberlindnera* [97]. Crosstalk between the virome and mycobiome, as well as the host genotype and phenotype including sex, age, and presence of co-morbid conditions, lifestyles such as diet, hygiene, and occupation can contribute to intestinal immune homeostasis [98].

The mycobiome and virome work in synergy with the bacteriome community of the microbiota to modulate the host immunity and physiology. However, limited data have been published concerning the virome-mycobiome crosstalk. Evidence has shown that a tight equilibrium exists between the mycobiome, host, and other microbiome entities which help in maintaining tissue equilibrium. Most specifically, *C. Albicans* contributes to the recolonization of the intestine by bacterial species (*Bacteroides*) after antibiotic treatment [99]. Mono-colonization of the intestine with *C. albicans* or *S. cerevisiae*, fungi species that are widely recognized by CX3CR1+ MNPs, supports the establishment of intestinal homeostasis and protects against virus-induced lung inflammation and DSS-induced gut barrier damage [100]. Intestinal viruses and fungi have been seen to have extra-intestinal effects on the host immunity, by modulating systemic immune responses as seen in patients with type 1 diabetes and in NOD mice [101].

Limited data have been reported to demonstrate the interkingdom interaction between the virome and mycobiome. However, different alpha and beta diversity of the salivary mycobiome has been observed in individuals with viral infections such as HIV infected individuals [102]. Nonetheless, to date, only a few studies have addressed the interaction between fungi and viral component of the microbiome and how it affects the host. For this review, the limited amount of information is discussed using three main approaches, mycobiome interaction with eukaryotic viruses, mycobiome interaction with prokaryotic viruses, and possible interaction with human endogenous retroviruses as it affects immune homeostasis and diseased states.

### 6.1. Mycobiome Interaction with Eukaryotic Viruses

Recent finding sheds lights on the complex interkingdom interactions between viruses, fungi, and other members of the microbiota [103]. Suggested pathways of viral effects on the mycobiome had possibly been through inflammatory conditions created by host in response to viral infections [104, 105]. Such sequela events which include swarms of inflammatory cells, modulation of receptor expression, damaged epithelia barrier and rapid turn-over, presence of growth factors, and other cytokine-rich environment could serve as triggers for fungal growth. However, Plotkin and colleagues [103] successfully demonstrated these viral-fungi interactive pathways at least preliminarily. Using *in vitro* infection of HeLa cell culture with HSV-1 and HSV-2, they revealed distinct morphological growth and adherence of *Candida albicans* while simultaneously inhibiting the adherence of *Staphylococcus aureus*. The *C. albicans* and *S. aureus* are both commensals occupying distinct anatomical locations in the body [103]; however, colocalization occurs in diseased, immunocompromised states or when surfaces that promotes biofilm formations are available, e.g., catheters and feeding tubes. One factor possibly responsible for their most often mutually exclusive adherent sites in healthy states could be from the affinity of *S. aureus* to sulfated heparans abundant on epithelia cells which aid biofilm formation [106–108]. Antagonistically, such heparan derivatives block *Candida* attachment to cell surfaces thus preventing biofilm formation [109]. However, following HSV virus cellular entry by endocytosis, several reports have shown that there is a downregulation of sulfated heparans molecules which is detrimental to *S. aureus* adherent mechanisms and preferentially favors *C. albicans* fastidiousness. Such virus-host cell-fungal interaction was suggestively reported responsible for the results of the experiments of Plotkin et al. stated above. One notable exception from the results however was the induction of yeast forms of *C. albicans* by HSV-1 strain and a more pathogenic filamentous form by HSV-2 strain. However, the precise mechanism needs further research. Furthermore, Cermelli et al. had earlier showed that macrophages infected with HSV portray dysfunctional phagocytic ability of Candida which stemmed from altered gene expression events and dysregulated oxidative bursts, thus promoting Candida survivability [103, 110]. Additionally, the repertoire of the mycobiome is reported to have potent anti-inflammatory properties. For example, studies have confirmed the survivability of HSV virus in *C. albicans* biofilms is due to both decreased accessibility of antivirals to HSV and also the anti-inflammatory environment induced by *C. albicans* [103, 111]. Candida has also been reported to be abundant in HIV-positive individuals, but without a statistical difference from HIV-negative persons [102]. Furthermore, increased abundance of adenoviruses and anelloviruses has been reported in fecal samples of HIV-positive individuals with low CD4+ T-cell counts [112].

### 6.2. Mycobiome Interaction with Prokaryotic Viruses

Similar to the relationship with eukaryotic viruses, much of direct fungi-bacteriophage interactions or any other member of the prokaryotic virome family remains to be explored with few studies highlighting this relationship and possible potential benefits. Mycophages or mycoviruses are members of the phage family that infects fungi [113]. They are mostly double stranded RNA viruses with few exceptions that are single-stranded RNA belonging to the family *Partitiviridae*, *Narnaviridae*, and *Totiviridae*. Most mycoviruses are found in fungi families that infects plants but have members that are pathogenic and mostly opportunistic in small animals and humans [114]. Mycome-mycophage relationship could span extremes of spectrum ranging from beneficial, cryptic, or harmless to pathogenic phenotypes. An example of the latter interaction has been documented for double-stranded RNA viruses and *Saccharomyces cerevisiae* yeast with regard to toxin secretion in specific phenotypes [115]. Although *Saccharomyces cerevisiae* is commonly found in environment, interactions with the mycoviruses and diets are sources by which *Saccharomyces cerevisiae* becomes part of gut microbiome [97] and under certain conditions could become pathogenic. Other toxic form of yeast has also been described with dsRNA mycovirus encoded toxin or encapsulation of toxic secretions such as found in some members of the family *Totiviridae* [116]. Beneficial properties of fungi-mycoviral relationship are well documented for a few fungi examples. For example, with regard to interferon inducing properties of cultured *Penicillium* genus in animals, dsRNA mycoviruses have been strongly linked to this property which has spurred research interests along this field [115, 117]. Although not directly related to the gut, a predominantly well-studied model of such interkingdom interaction is seen in the case of cystic fibrosis in the lungs where phage Pf4 from bacteria *Pseudomonas aeruginosa* strain PA01 inhibited the growth of *Aspergillus fumigatus* [118, 119]. This inhibitory mechanism elicited by filamentous phage of genus Inovirus was due to sequestration of ferric ion (Fe^3+^) which is vital to survival of *A. fumigatus* and thus the severity of the disease [118, 120, 121]. Fungi-growth inhibitory property following Fe^3+^ sequestration was also found effective against *Candida albicans* and other species and abolished in presence of supplemental iron administration [122]. This relationship opens a vista of opportunities to therapeutic exploration of the understanding of fungi-virome relationship in the treatment of gut-related disease such as inflammatory bowel syndrome where potent anti-inflammatory properties of mycobiota would be additional benefits.

### 6.3. Mycobiome Interaction with Endogenous Retroviruses

Studies are very sparse when it comes to relationship between human endogenous retroviruses (HERVs) and the mycobiome. However, a possible link and area worthy of further studies might be the effect of HERVs on the mycobiome population in health and diseased states and vice versa. A baseline for such study comes from late twentieth century reports of insulin and insulin-like molecules in lower eukaryotic organisms such as worms, insects, bacteria, and fungi, e.g., *Aspergillus fumigatus* and *Neurospora crassa* [123]. In fact, McKenzie et al. [124] successfully demonstrated an increased growth in morphology and metabolism of *Neurospora crassa*—a model organism when grown in presence of mammalian insulin. Although *N. crassa* is not reported as part of the microbiota, such effect might be present among members of the human mycobiome which needs further studies. Evidence for such proposed studies is buttressed from the results of Al Bataineh et al. where links between gut microbiome and fungal population were examined in type 2 diabetic patients and controls. In the diabetic groups, *Malassezia furfur* and an unclassified genus—*Davidiella*—were significantly associated with an increase in diabetic states while another unclassified genus—*Basidiomycota*—was found to be significantly decreased in diabetic group [125]. Further, Tsumura et al. reported increased expression of type-c retroviral particles in pancreatic *β*-cells of diabetic NOD mice with more severity and production of intra-cisternal A—particles when exposed to cyclophosphamide—a pattern absents in diabetic resistant mice [126]. This increased expression of HERVs in the development of pancreatic inflammation suggests a possible role for endogenous retroviruses in the diabetic pathogenesis from mice models. Such pathways were explored when Everard et al. (2014) demonstrated that administration of *Saccharomyces boulardii* changes gut microbiota population and eventually reduces fats accumulation in the liver, inflammation, and general fat mass in obese and type-2 diabetic mice models. Considering the unexpectedly strong anti-inflammatory roles reportedly played by fungi component of the microbiome vis a vis their small population [127, 128], possible links between changes in mycobiome phenotypic representation and trigger of HERVs expression in inflammatory states might exist which would be a subject of further research.

### 6.4. The Effect of Intestinal Helminth Infections on the Mucosal Immunity and Its Effect on Viral Pathogenesis

Helminths are parasitic worms that affect a variety of different host species. Epidemiological data suggest that over 2 billion people have been infected worldwide by parasitic helminths, especially in developing regions, such as sub-Saharan Africa, South America, and India [129]. The long co-evolutionary relationship between helminth infections and man is known to have a significant impact on immune responses to primary infection. In fact, the interaction between helminths and the host's immune system has been shown to provoke immunomodulatory and immunoregulatory mechanisms that ensure their survival in the host for years [130]. Emerging evidence also suggest that the establishment of chronic parasitic infections in endemic regions have significant implications on vaccine responses. Generally, the gut immune response in chronic parasitic infection is largely Th2 in nature. It is characterized by the activation of cells of the innate immune system such as dendritic cells (DCs), type 2 macrophages, regulatory T-cells (Tregs), regulatory B cells (Bregs), eosinophils, basophils, and mast cells. The recognition of helminth-associated PAMPs by these cells often results to the release of several cytokines such as interleukin (IL)-4, IL-5, IL-9, IL-10, IL-13, IL-21, IL-25, IL-33, and transforming growth factor (TGF)-*β* which have downstream effects on the CD4+ and CD8+ T-cells of the adaptive immune system [131–133]. Severe acute infections and a successful establishment of chronic infection by most intestinal helminth parasites have been shown to favor the pathogenesis of most viruses that infect the gut. Perhaps, intestinal helminths are known to generate strong T helper 2- (Th2-) driven cytokine responses, which counter the biological effects of IFN-*γ* (important for Th1 responses), and also polarize M1 (pro-inflammatory) macrophages towards the M2 (immunoregulatory) phenotype [6, 134]. A recent study demonstrated the exaggeration of vaginal HSV-2 pathology following acute infection with *Nippostrongylus braziliensis* in mice models [7, 135]. Results from this study showed that mice infected with *Nippostrongylus braziliensis* induced a type 2 immune profile in the female genital tract. This triggered eosinophil recruitment and promoted an eosinophil, IL-33, and IL-5 inflammatory circuit that enhances vaginal epithelial necrosis and pathology following HSV-2 infection of the female genitalia [135]. This result was further confirmed by treating mice with the *α*-Siglec-F antibody to deplete them of eosinophils prior to the virus infection. The eosinophil depleted co-infected mice displayed rescued pathology equivalent to HSV-2-only infected mice [135]. Another report from Peru showed that women in helminth-endemic regions had an increased risk of human papillomavirus (HPV) infection compared to those in non-endemic regions. In fact, the prevalence of HPV was seen to be higher among the former group compared to the latter [136]. As previously indicated, infection with intestinal helminths can alter the biological functions of some cytokines that are crucial for the induction of a potent Th1 response which is known to trigger protectivity against viral infections. This hypothesis has been tested in series of experiments involving mice models. It was shown that mice infected with the intestinal helminth, *Heligmosomoides polygyrus*, were able to induce the reactivation of latent murine herpes virus 68 (MHV68) infection [137]. The helminth infection was characterized by the induction of the cytokine interleukin-4 (IL-4) and the activation of the transcription factor STAT-6, which reactivated the murine gamma herpesvirus infection *in vivo*. The helminth-induced IL-4 was shown to enhance viral replication and blocked the antiviral effects of IFN-*γ* by upregulating the viral latent-to-lytic switch gene (*gene 50*). This is because of the IL-4-activated STAT-6 which promotes viral replication by binding to and acting on the viral promoter necessary for the expression of gene 50 [137]. Thus, chronic infection due to herpesvirus which is a component of the mammalian virome can be regulated through the counterpoised actions of multiple cytokines on viral promoters that have evolved to sense host immune status. Several studies have elucidated the inverse relationship between intestinal helminth infections and viral pathogenesis, with most reporting an exaggerated outcome on the viral pathogenesis. However, there is still controversy on whether this inverse relationship exists for helminth and HIV coinfected patients. It should be noted that HIV is a major component of the human virome, and it has been shown to co-evolve with man for several decades. Some immunological data suggest a range of scenarios in which intestinal helminths and HIV may each either promote or oppose acquisition or progression of the other condition. Like other viral co-infections, helminth-induced immuno-regulatory mechanisms can impair protective responses to HIV [138]. Although this seems to always be the immunological scenario, other studies have indicated a beneficial outcome from this immuno-regulatory mechanism. Perhaps, evidence has shown that the replication of pro-viral DNA depends on the activation of host cell transcription factors and helminth-induced regulatory activity can suppress such transcription [139]. This could therefore be beneficial especially in the context of HIV progression [140]. Reports have shown that *in vitro* human FoxP3 transduced Treg cells expressed high levels of the HIV coreceptor (CCR5) and are readily infected by HIV [141]. These cells are preferentially eliminated by direct HIV infection leading to uncontrolled immune activation and dysfunction. The high foxp3 expressing Treg cells have been found to correlate inversely with markers of immune activation [138, 141]. Thus, an increasing loss of these cells may reduce suppression of immune activation which might have some important implications for the host–parasite interaction. *In vivo* experiments with animal models of immunosuppression suggest that granuloma formation and egg excretion by *Schistosoma mansoni* might be reduced in HIV infection [14, 142]. Moreover, studies in humans supported this hypothesis with evidence of reduced egg excretion in HIV-infected subjects [143–145]. Considering the already existing evidence on the immunoregulatory mechanism induced in most helminthic infections and their effect on viral pathogenesis, it would be imperative to have a detail understanding on this complex interplay between the immune system and helminths. In fact, a critical understanding of the interplay between parasites and the microbiome and its role in the pathogenesis of viruses will be important, also in light of future application of vaccine programs as well as therapeutic strategies.

## 7. Role of Virome in Intestinal Disease

### 7.1. Role of the Virome in Inflammatory Bowel Disease

Inflammatory bowel disease (IBD) which encompasses Crohn's disease (CD) and ulcerative colitis (UC) is an inflammatory disorder characterized by chronic inflammation of the intestinal tract with periodic flares and remissions. Even though a lot of research is still needed to understand the etiology of the condition, what remains clear is that it is multifactorial and has a close association with an altered microbiome in the human gut, i.e., a reduced diversity in the bacteriome, particularly a drop in the population of the Firmicutes and *Bacteroides*. However, there is a growing body of evidence that seems to highlight the association of an altered gut virome and IBD [51, 146, 147]. A wide range of viruses which include eukaryotic viruses, bacteriophages, and a certain number of viruses such as Epstein Barr virus (EBV) and cytomegalovirus (CMV) have been thought to influence the pathology of IBD through mechanisms that are still not clear [148, 149]. Noroviruses for instance has been shown in murine model to suppress a lot of the beneficial functions of symbiotic bacteria after transplantation which might possibly contribute to the progression of IBD [16]. Also, it has been shown in another study that certain viruses can have an effect on the microbial diversity in the gut [16]. Viruses can break the tolerance to bacteria in CD patients and show a co-variation with bacterial strains [150]. A lot of these viruses are bacteriophages which under normal conditions play an important role in maintaining homeostasis in the microenvironment and by serving as transmitters to deliver genetic material to bacterial communities. Alteration in the population of the enteric bacteriophages can therefore significantly change the bacterial fitness and result in gastrointestinal diseases [151, 152].

Recent studies have pointed to an increase in the abundance of the bacteriophage family of Caudovirales as the most significant alteration in the virome associated with IBD [153]. The five Caudovirales family of bacteriophages identified by Norman et al. includes the *Clostridium*, *Enterococcus*, *Lactococcus*, *Lactobacillus*, and *Streptococcus* bacteriophages. Analysis of some bacterial taxa associated with IBD also revealed there exists an inverse correlation between bacterial diversity and alterations in the Caudovirales bacteriophages [51]. A plausible explanation for this might be that the activation of latent prophages results in the lysis of their host bacterium and may further set up a downstream inflammatory signaling to cause the release of cytokines, infiltration of cells, and eventually tissue damage [154]. Also *in vitro* studies have shown that bacteriophages can be recognized by the innate immune system and induce inflammation through the production of My-D88-dependent proinflammatory cytokines [155]. Although most research has been concentrated on phage virome, perturbations in the eukaryotic virome have also been associated with the pathogenesis of IBD [27]. Using deep sequencing techniques to decipher alterations in the gut virome, it was highlighted by Zuo et al. that patients with UC showed an increased abundance of Pneumoviridae as compared to the control while the reverse was observed for the Anelloviridae family [147]. In a study that also analyzed colon samples of IBD patients as against control patients also revealed the heightened levels of the *Herpesviridae* family as well as an increase in the expression of endogenous viral sequences [156]. To further elucidate this association, larger studies would be needed even though the role of some herpesviruses in the development and exacerbation of IBD has already been described [157]. A more recent study by Ungaro et al. through a metagenomic analysis has shown an increase in the abundance of *Hepadnaviridae* family in UC patients. However, *Polydnaviridae* and *Tymoviridae* viral families which are associated with diet were less found in patients with UC with similar observation for *Virgaviridae* in CD patients [158]. The drawback with these studies has been that findings have been drawn from compositional changes from the fraction of the virome that could be identified which constituted about 15% of the sequence data of the virome. In a study that reanalyzed existing data in a data-independent manner, the authors showed that a core virome in healthy individuals shifts to a less stable community that is dominated by phage in IBD. The study also highlighted the fact that the changes in the virome in IBD is accompanied by changes in the bacteriome and that a combined assessment might serve as a better method for classifying IBD patients from healthy subjects [153]. In light of the evidence gathered so far, it can be concluded that the gut virome could potentially contribute to the IBD pathogenesis by inducing a dysbiosis from its interaction with the bacteriome through microbial lysis, epithelial cell infection, or direct immune activation following translocation through the epithelial cells [159]. With altered virome likely to play a role in the pathogenesis and progression of IBD, it has also become important for clinicians to investigate the potential risks with the use of glucocorticoids and other immunosuppressive agents to avoid the risks of serious of viral infections that comes with immunomodulation. Patients under treatment for IBD usually have opportunistic infections such as CMV, EBV, herpes viruses, and human papilloma virus (HPV) [19, 160]. Even though the exact mechanism of pathogenesis is not known for these viruses, there is evidence to suggest they can influence the progression of IBD. This is accompanied by relatively high mortality and morbidity rates for patients whose immune system has been compromised [161].

### 7.2. Irritable Bowel Syndrome

In a metagenomic sequencing study of the Fecal Virus-like Particles in Irritable Bowel Syndrome (IBS) Patients and Controls by Coughlan et al., the authors demonstrated an alteration in the virome of patients. IBS is one of the most commonly diagnosed gastrointestinal disorders, mostly associated with alterations in the bacteriome. However, it was revealed that IBS was associated with a reduction in alpha diversity of both novel and known viruses as well as a significant difference in beta diversity [162]. Furthermore, they showed that bacteriophage clusters belonging to the order Caudovirales (Siphoviridae, Myoviridae, and Podovirdae) were the most abundant [162].

### 7.3. Diarrheal Diseases in Children

Diarrheal diseases in children seem to demonstrate an important contribution of the host intestinal virome. Next generation sequencing-enabled metagenomic studies have enabled the identification of known and previously unknown viruses as the etiological agents of these diarrhea diseases. The newly named viral families Bufavirus, Picobirnavirus, and Pecoviruses have been detected and characterized in separate studies in the stools of children with diarrhea of unknown etiology [163]. The gut virome analysis has also led to the identification of viruses that have not been previously shown to be linked to pediatric diarrhea and gastroenteritis such as Picobirnavirus, Anellovirus, and Smacovirus [164, 165].

### 7.4. Celiac Disease

Celiac disease is an autoimmune enteropathy induced by gluten ingestion which has so far been shown to have a significant genetic predisposition. However, additional environmental factors have been suggested to be involved in the pathogenesis of celiac disease. Several studies have pointed to a possible role of viral infections particularly from Adenovirus, Rotavirus, and Reovirus in the pathogenesis of celiac disease [163]. Moreover, screening of fecal virome in a metagenomic study also revealed an association between Enterovirus infection and the risk of celiac disease (a subclinical or preclinical phase of celiac disease) [166].

### 7.5. The Role of Virome in Cancer of the Large Intestine

Colorectal cancer is known to be one of the most frequent causes of cancer-related death in Europe and second most common in the USA. Several risk factors have been described, which include genetic predisposition, diet, and environment. In recent years, studies have been geared towards investigating the role of the gut microbiome in the pathophysiology of colorectal cancer [167–169]. Of the well-studied microbiome population in the gut, the bacteriome has received much attention with fewer emerging studies about the virome and their role in development of cancers of the large intestine [Stulberg et al., 2016; Zou et al., 2016; Delwart et al., 2013]. Cancers of the colon and rectum arise from the epithelium which has a high turnover rate (about 10^10^) every 2-5 day and is in constant contact with the luminal microbiota [170]. The roles of bacteriome—the most abundant and characterized among the gut microbiota population in relation to colorectal cancer—have been clearly elucidated in literature [171–173]. In fact, characterization of the bacteriome population in the gut is being employed as diagnostic tools in the classification of healthy, dysbiosis (adenomatous), and cancerous colon [174, 175]. However, until recently, little information is known regarding the role of the virome in the pathophysiology of colorectal cancer and their potential diagnostic applications. This limitation of knowledge and potential applications as hitherto being due to lack of precise molecular diagnostic methods to characterize and investigate their functions [176] as well as identifying the exact taxonomic phyla that these viruses belong. Within the last few decades, the significant advancement in scientific research has made it possible for scientists to classify some of these viruses and decipher their role in host homeostatic condition, contribution to inflammatory disease states in the gut, and different stages of colorectal cancer [177].

The influence of the virome in the development of diseased states in the gut can be classified as a direct or indirect effect. The direct role originates from the effect of individual gut-dwelling viruses associated with disease conditions. For example, the toroviruses, coronaviruses, caliciviruses, adenoviruses, picornaviruses. from the Eukaryotic family and specifically Polyoma JC virus (JCV) and human papilloma viruses [170] which could trigger or contribute to the development of colorectal cancers with or without other risk factors [178].

The JCV, which is a double-stranded DNA virus, is known to have a predilection site for the kidneys and infects about 80% of people with symptomatic diseases such as progressive multifocal leukoencephalopathy (mostly associated with immunosuppressive events). Several studies have identified the JCV genome in 30% of normal, 60% of adenomatous, and 61% in cancerous colon tissues with an odd ratio of 6.2% (at 95% confidence interval) [170, 179–181]. The viral genome copies also have statistically significant higher numbers in cancerous colon compared to the normal ones [170]. In terms of the mechanism of oncogenic induction, it has been shown that JVC large T protein antigen induces a G_0_ cell into S-phase by interacting with the cell cycle control proteins such as p53 as well as tumor suppressor protein pRb, thereby resulting in uncontrolled cell division (cancer) [178, 182]. It also activates a downstream substrate of insulin-like growth factor I receptor (IRS) prompting a cellular proliferation and survival signals through the PI3-K pathway [182–184]. Emerging evidence has shown that the JCV large T antigen can directly predispose cells with IRS 1 gene polymorphism to cancer. This further highlights the role of latent JCV in susceptible individuals or in cancer progression (Virol J, 2010). In connection to this, JCV in susceptible individuals or non-immunocompromised patients can interrupt with the DNA repair mechanisms through altered expression of the Ku70 and Ku80 repair proteins [185, 186]. As a consequence, this results to the stabilization of the *β*-catenin that is involved in Wnt-pathways, which activates c-myc and cyclin D genes in a sequence of downstream signaling events and promotes cellular proliferation [179, 187]. With regard to *in vitro* model of colon epithelia cells, studies by Ricciardiello et al. have demonstrated that these genetic mutations caused by JCV lead to instability. These disruptive activities of JCV associated with initiation of uncontrolled proliferation of cancerous cells with various degree of phenotypes occurring in the intestine could lead to dysplasia, trigger the release of alarmins and stress factors that results in an inflammatory microenvironment, and recruitment of innate immune cells with subsequent activation of the adaptive immune response. Although there are many neurotropic strains of JCV, only 98 base pair deficient Mad-1 strain has been associated with colorectal cancer [188].

Also, there have been reports, although somewhat conflicting, about the role of human papilloma virus (HPV) (the leading cause of cervical cancer) in colon cancers [170]. Most studies however agree a possible role for HPV in colon cancer. The exact mechanism of HPV induction of colorectal cancers is still to be elucidated but reports have emerged about papillomavirus associated colon cancer without p53 mutations—a common phenomenon on cervical cancer cells [189–191].

The indirect role however depends on the bacteria population present in the gut at any given time. The most common bacterial population of the microbiota identified is *Bacteroidetes*, Firmicutes, *Actinobacteria*, and *Proteobacteria* in decreasing other. Conversely, aside the commonly known pathogenic viruses such as rotaviruses, enteroviruses, and norwalk viruses, which could cause prolonged gastroenteritis in man, prompting microflora changes that induces GI disorders as in IBD, other viruses such as giant viruses, plant-derived viruses, and bacteriophages have been described [33].

## 8. Colitis-Associated Cancers

First coined by Greten et al. to describe the role of NF-*κ*B in persistent inflammatory cycle leading to cancer, numerous studies have elucidated the role of chronic inflammation and mechanisms by which cancer develops in these environments [192, 193]. As the cascade (Figure 2) begins with viromes, perpetuated by risk factors and pathogenic bacteria, PAMPS from the latter signaling through the TLR 2/4-MYD88-NFKb pathway results in the production of inflammatory cytokines such as IL-1, TNF-*α*, and IL-6. Influx of neutrophils and macrophages also propagates the inflammatory reactions by releasing IL-8, Il-6, IL-12, and TNF-*α* [194]. Macrophage-derived IL-6 has been reported to engage the IL-6R on epithelia cells, which alongside the gp130 signals to induce STAT3 and in turn propagates inflammatory cytokines production through retention of RelA component of NF*κ*B in the nucleus. STAT3 also induces cellular proliferation through directly interacting with cell cycle regulator [194]. Similarly, studies have implicated IL-6 in the downregulation of p-53 family of genes thereby allowing cell division events unchecked [195]. Lastly, the effect of TNF-*α* on the virome-bacteriome-risk factor mediated inflammatory environment have been described as a vital link into cancerous states. TNF-*α* is produced by T-cells and myeloid cells recruited to the inflammatory site and acts on TNFR1 or TNFR2. The latter receptor is expressed on intestinal epithelial cells and is involved in the activation of the NF-*κ*B pathway, with subsequent release of kinases that degrades myosin and break junctional complexes—thereby activating a vital step in dysplastic growth and early tumorigenesis [196, 197]. Put together, these drivers—IL-6, TNF-*α*, and IL-17 family of cytokines—looks central to the progression of inflammatory states triggered by the virome, aided by the bacteriome and risk factors that can result to the development of cancers in the large intestines.

### 8.1. The Use of the Virome as Future Therapeutics

The use of the gut microbiome as therapeutic targets has been extensively explored in the treatment of several human diseases like inflammatory bowel diseases, infectious diseases, and others [198]. Although most of these studies are focused on the bacteriome, other studies have been to unravel, particularly the therapeutic potential of some prokaryotic viruses (bacteriophages) in quest for the development of effective vaccines and immunotherapies against infectious diseases and certain tumors [199]. Several approaches have been adopted concerning the development of phage-based vaccines. For instance, some studies have been geared towards displaying an antigen of interest as a fusion protein on the capsid surface [200] or directly conjugating the antigen to the surface of the phage without altering the genome. As described by Krystina L. et al. (2019), it has been shown that B6 mice immunized with OVA-peptides expressed on filamentous phages displayed significantly lower levels of blood stage and myocardial parasitemia compared to control mice after challenged with OVA expressing *T. cruzi* [199]. This suggests a possible induction of an antigen-specific immune response, which was capable of protecting mice from the *T. cruzi* infection.

Furthermore, phage-based therapy has also been explored in the context of some respiratory viral infections such as the influenza A virus that is known to favorably infect the respiratory epithelium. In connection to this, studies have shown that the infection of the epithelium by pathogenic microbes often results from the interaction with M cells through the expression of invasin surface protein that binds to *β*1 integrins on the M cell surface. Recent studies have been geared towards harnessing this interaction for the development of effective phage-based vaccines against mucosa infecting pathogens. Moreover, it has been shown that the infection of invasin-expressing *E. coli* cells with filamentous phage (engineered to express the highly conserved matrix 2 protein of influenza A virus) resulted in the accumulation of the infected *E. coli* cells within Peyer's patches following oral administration in mice. Furthermore, the authors revealed that after successive oral administrations of the phage-infected *E. coli* cells, the mice developed M2e-specific IgG antibodies, which protected them from a sub-lethal dose of mouse-adapted influenza A [201].

Compelling evidence shows that phages can also be used as vehicles for vaccination. Perhaps like other vaccine delivery molecules such as nanoparticles, phage particles expressing an antigen of interest can also be recognized as foreign and taken up by antigen presenting cells (APCs) [202]. This approach has been applied in the context of cancer therapy. It has been reported that intra-tumoral injection of tumor bearing mice with the filamentous phage expressing the antigenic determinant of OVA resulted in the induction of antigen-specific T cell response, which delayed further tumor growth and increased survival [202]. Furthermore, some oncolytic viruses (OVs) (e.g., Adenovirus, vaccinia virus, HSV, reovirus, and measles virus) belonging to part of the human eukaryotic virome have been explored for the development of effective cancer therapy. This therapy is based on breaking the tolerogenic tumor microenvironment and subsequent stimulation of antitumor immunity [24]. The oncolytic viruses are designed to target cancer cells without causing any damage to the normal cells. Perhaps, the entry of the OVs to their targets (cancer cells) is dependent on multiple factors including the presence of cell surface receptors necessary to facilitate virus binding/entry, metabolic status of the cell, and the ability of the virus to overcome the intracellular innate immune or antiviral downstream signaling pathways within the cancer cells [203]. Several OVs-based therapies have been approved for the treatment of certain cancers. For example, the FDA approved T-VEC (Imlygic) for the treatment of melanoma in 2015 [204]. T-VEC is a modified form of HSV-1 with deletion of specific genes that favors their selective replication within the cancer cells, with subsequent increase in presentation of viral and tumor antigens [205]. Also, the State Food and Drug Administration of China in 2005 approved Oncorine (a genetically modified type 5 human adenovirus (HAdV-C5) with deletion of the E1B-55KD and E3 regions to induce selective replication in p53 defective cells and increase safety) for the treatment of head and neck squamous cell carcinoma [206].

Another approach such as the fecal virome transplantation (FVT) is continuously being explored for the treatment of certain disorders. Results from in vivo studies with murine models showed that FVT from donor lean mice led to a reduction in weight gain and normalized glucose tolerance in obese recipients [207].

The successful preclinical and clinical results already achieved with phage-based therapy, oncolytic virotherapy, or FVT provide clear indications on the prospected advantage of the virome as effective therapeutic tools against certain cancers and infectious diseases.

## 9. Conclusion

The virome indeed has a central role as a crucial determinant of individual state of the gut in terms of health and diseases. The trans-kingdom interaction with other members of the microbiome is a key in shaping the intestinal microbial population which could be beneficial on one hand and predispose to dysbiosis on the other hand. These complex inter-phylogenic interactions ensure that the virome may be able to modulate or influence bacterial population and colonization with indirect effects on the immune tolerance mechanism or directly influence the homeostatic balance in the gut. Indeed, the metagenomics of the virome needs further studies to elucidate the protective metagenome signatures with the development of novel strategies to exploit the virome for therapeutic applications. In this regard, the bacteriophage has been promising as bacteriophage therapy is gaining popularity in clinical applications for addressing dysbiotic states and antimicrobial resistance. Furthermore, future studies to have a broader elucidation of the modulatory role of bacteriophage and the ERVs in IBD and colon cancer may open doors for more precise therapeutic approaches to address these disease conditions from the perspective of targeting a homeostatic microbial composition. For example, diagnostic capabilities can range from employing phage specific identification in the diagnosis of ulcerative colitis and Chron's disease using fecal samples in situations where there is overlap in bacterial population or indistinguishable clinical presentations. In addition, the use of prebiotics and probiotics can be modified in a way that targets the maintenance or induction of a specific desirable phage-population which can in-turn prevent disease induction or modulate its course. Overall, there is need for more studies into the virome metagenomics and trans-kingdom interactions for full characterization and potential exploitation in understanding intestinal disease pathogenesis and therapeutic applications.

## Figures and Tables

**Table 1:** Different body sites and the associated virome. A summary of the families of phages and eukaryotic viruses distributed at different sites in the human body (adapted from Liang and Bushman, [46]).

Sites	Phages	Eukaryotic viruses
Blood	*Siphoviridae*, *Podoviridae* *Myoviridae*, *Microviridae* *Inoviridae*	*Anelloviridae* *Herpesviridae* *Picornaviridae*
Vagina	*Siphoviridae*, *Podoviridae* *Myoviridae*, *Microviridae*	*Anelloviridae*, *Herpesviridae*
Oral cavity	*Siphoviridae*, *Podoviridae* *Myoviridae*	*Herpesviridae*, *Redondoviridae*, *Anelloviridae* *Papillomaviridae*
Skin	*Siphoviridae*, *Podoviridae* *Myoviridae*	*Adenoviridae*, *Anelloviridae* *Circoviridae*, *Herpesviridae* *Papillomaviridae*, *Polyomaviridae*
Urinary system	*Siphoviridae*, *Podoviridae* *Myoviridae*	*Papillomaviridae*, *Polyomaviridae* *Herpesviridae*
Lung	*Siphoviridae*, *Podoviridae* *Myoviridae*, *Microviridae* *Inoviridae*	*Anelloviridae*, *Redondoviridae*, *Adenoviridae*, *Herpesviridae* *Papillomaviridae*
Gastrointestinal tract	*Siphoviridae*, *Podoviridae* *Myoviridae*, *Microviridae* *Inoviridae*	*Anelloviridae* *Adenoviridae*, *Caliciviridae* *Picornaviridae*, *Herpesviridae*, *Circoviridae* *Virgaviridae*

**Table 2:** Examples of intestinal viruses and their role in maintaining gut health.

Virus	Mechanism	Outcome	References
*Caudovirales*	Colonization of donor-derived Caudovirales taxa in recipients.	Effective treatment of recurrent *C. difficile* after fecal microbiota transplant (FMT)	[35]
Lymphocytic choriomeningitis virus (LCMV)	Infects and replicate in lymphocytes, thereby disordering their function and favoring immune suppression	Prevention of type I diabetes in NOD mice	[47]
Murine gammaherpesvirus 68	Prevents the development and progression of autoimmune lupus-like disease in mice	Inhibits the activation of T cells, B cells, and dendritic cells.	[48]
Murine cytomegalovirus	Latent infection protects host from intracellular bacterial infection such as *L. monocytogenes*	Latent infection triggers elevated levels of IFN-*γ* and TNF-*α* by activated macrophages	[49]

## Data Availability

All data included within the article.
